# Combination of problem-based learning with high-fidelity simulation in CPR training improves short and long-term CPR skills: a randomised single blinded trial

**DOI:** 10.1186/s12909-019-1626-7

**Published:** 2019-05-31

**Authors:** Christian Berger, Peter Brinkrolf, Cristian Ertmer, Jan Becker, Hendrik Friederichs, Manuel Wenk, Hugo Van Aken, Klaus Hahnenkamp

**Affiliations:** 1 Department of Anesthesiology and Operative Intensive Care Medicine, Charité-Universitätsmedizin Berlin, Campus Benjamin Franklin, Hindenburgdamm 30, 12200 Berlin, Germany; 2 Department of Anesthesiology, University Medicine Greifswald, Greifswald, Germany; 3 Department of Anaesthesiology, Intensive Care and Pain Medicine, University Hospital Münster, Münster, Germany; 4 Institute for Education and Students Affairs-IFAS, Medical Faculty, University of Münster, Münster, Germany

**Keywords:** Cardiopulmonary resuscitation, Medical students’ education, Problem-based learning, High-fidelity simulation, Hands-on training, Advanced adult CPR

## Abstract

**Background:**

Performance of sufficient cardiopulmonary resuscitation (CPR) by medical personnel is critical to improve outcomes during cardiac arrest. It has however been shown that even health care professionals possess a lack of knowledge and skills in CPR performance. The optimal method for teaching CPR remains unclear, and data that compares traditional CPR instructional methods with newer modalities of CPR instruction are needed. We therefore conducted a single blinded, randomised study involving medical students in order to evaluate the short- and long-term effects of a classical CPR education compared with a bilateral approach to CPR training, consisting of problem-based learning (PBL) plus high fidelity simulation.

**Methods:**

One hundred twelve medical students were randomized during a curricular anaesthesiology course to a control (*n* = 54) and an intervention (*n* = 58) group. All participants were blinded to group assignment and partook in a 30-min-lecture on CPR basics. Subsequently, the control group participated in a 90-min tutor-guided CPR hands-on-training. The intervention group took part in a 45-min theoretical PBL module followed by 45 min of high fidelity simulated CPR training. The rate of participants recognizing clinical cardiac arrest followed by sufficiently performed CPR was the primary outcome parameter of this study. CPR performance was evaluated after the intervention. In addition, a follow-up evaluation was conducted after 6 months.

**Results:**

51.9% of the intervention group met the criteria of sufficiently performed CPR as compared to only 12.5% in the control group on the day of the intervention (*p* = 0.007). Hands-off-time as a marker for CPR continuity was significantly less in the intervention group (24.0%) as compared to the control group (28.3%, *p* = 0.007, *Hedges’ g* = 1.55). At the six-month follow-up, hands-off-time was still significantly lower in the intervention group (23.7% vs. control group: 31.0%, *p* = 0.006, *Hedges’ g* = 1.88) but no significant difference in sufficiently performed CPR was detected (intervention group: 71.4% vs. control group: 54.5%, *p* = 0.55).

**Conclusion:**

PBL combined with high fidelity simulation training leads to a measurable short-term increase in initiating sufficient CPR by medical students immediately after training as compared to classical education. At six month post instruction, these differences remained only partially.

**Electronic supplementary material:**

The online version of this article (10.1186/s12909-019-1626-7) contains supplementary material, which is available to authorized users.

## Background

Surviving cardiac arrest is dependent upon the quality and timely initiation of optimal cardiopulmonary resuscitation (CPR) performed by medical personnel. [1] However, evidence has accrued that suggest a lack of knowledge exists amongst health care professionals regarding handling these time-critical situations, which can strongly influence the ultimate outcome for a patient experiencing cardiac arrest. [2–6] Enhancement in CPR education for medical students could improve CPR knowledge, performance, and outcome of CPR performed by later medical physicians. Therefore it seems to be reasonable to find the optimal way to teach CPR with a long as possible lasting effect.

Little is known about the most effective way of teaching medical students CPR and the half-life of CPR-skills after a training session may be briefer than previously assumed. [7–9] There is evidence to suggest that, higher self-confidence in performing CPR is associated with higher quality in basic life support (BLS) as measured via compression depth, compression frequency and manual ventilation. [10] Therefore, CPR training focussed on the acquisition of sustained skills in combination with improved self-confidence in initiating CPR may be beneficial.

Classical CPR training methods include theoretical lectures and instructor-guided CPR hands-on-training. In recent years, CPR simulation manikins have been developed as a high-fidelity simulation which are increasingly available in medical students’ education. It is unclear as to whether or not new training methods and up-to-date simulation equipment in realistic environments do contribute to improved CPR quality and outcome. [11–14]. Furthermore, it is unclear whether this has a measurable effect on long-term CPR performance.

Problem based learning (PBL) is an instructional learner-centered approach that empowers learners to research, integrate theory and practise and apply knowledge and skills to develop a viable solution to a defined problem [15]. This seems to be a promising alternative to classical instructor-guided CPR-training. There is evidence that graduates of PBL curricula demonstrate equivalent or superior professional competencies compared with graduates of more traditional curricula [16], but it is still unclear, if PBL leads to long lasting superiority of theoretically and practically skills, which seems to be a necessary combination for optimal performed CPR.

This raises the question, whether PBL teaching approaches combined with up-to-date manikin-technology make teaching CPR more effective. Furthermore, it is unclear whether this has a measurable effect on long-term CPR performance. In the present study we hypothesized, that PBL combined with a high-fidelity CPR simulation in a realistic environment improves short- and long-term CPR skills of medical students as compared to classical CPR-teaching techniques.

## Methods

We conducted a prospective, randomised, single-blinded interventional study involving fourth year medical students of the medical school of the University of Muenster, Germany. The study was approved by the ethics committee of the University of Muenster and the Medical Association of North Rhine-Westfalia (reference-number 2010–225-f-S). During a week long curricular anaesthesiology course combined with advanced life support (ALS) lectures and integrated CPR hands-on training, the students were randomized into a control and an intervention group by their matriculation number. Besides being student in the course named above, no specific inclusion or exclusion criteria were applied. The intervention and initial data collection took part in December 2010, data of the follow-up evaluation were collected in June 2011.

For blinding, all participants were informed that they would participate in a new educational approach, but neither group knew about the distinct study methods and purpose, nor about group allocation. In accordance with the ethic committee approval, information about the distinct study purpose and written informed consent was given after completed data collection. To avoid forwarding of information between students, we conducted the data collection in parallel in four separate rooms with four standard CPR manikins (Resusci Anne, Laerdal Medical GmbH, Puchheim, Germany) at the end of the course to minimize the risk for unblinding before data collection was completed. Apart from this, the students were asked to withhold this information until end of the data collection process.

A calculation of sample size was not performed, as this course was a mandatory part of the medical curriculum.

### Data collection by questionnaire

An initial paper-based questionnaire for empirical data collection and assessment of the participants’ self-perception of personal CPR-skills was completed. A second set of questionnaires was completed after the end of each educational unit, covering the training and time-dependent self-perception of the students’ CPR-skills (see Additional file 1). Each question had to be answered using a Likert-scale from 1-worst to 6-best.

### Teaching techniques

During the described curricular course, all participants initially received a 30-min theoretical lecture about advanced adult CPR according to the ERC guidelines [17]. In the following four days, practical and theoretical teaching was conducted in groups of six to eight students. Each group was allocated to one of four trained tutors. All tutors were experienced in BLS and ALS teaching and had been previously instructed in the PBL-tutorial process according to the PBL guidelines [18]. The tutors were not blinded and did not participate in data collection and analysing. To avoid tutor influence, tutor distribution among intervention and control group changed daily during the course.

The control group received classical CPR training. Classical CPR training was defined in this study as two different hands on advanced CPR scenarios on a standard CPR manikin (Resusci Anne, Laerdal Medical GmbH, Puchheim, Germany) in groups of 6 to 8 students, each lasting 45 min without further simulated environment. One tutor was present in each scenario, initially explained practically aspects and guided the students permanently for correct performed CPR. The tutor was allowed to interrupt the students and directly corrected and explained dubieties and errors. In each group, every student had to perform hands-on training and a feedback was given.

Participants in the intervention group first received a 45-min PBL-module followed by a 45-min CPR hands on training (*n* = 32) or vice versa (*n* = 26). The hands on training was conducted on a high-fidelity full-scale simulator (SimMan 3G, Fa. Laerdal Medical GmbH, Puchheim).

In the PBL-module, groups of 6 to 8 students had to solve a standardised advanced adult CPR-case in meeting the criteria for PBL-standard. [18] According to the PBL-tutorial process, the tutor guided to identifying and defining the problem and ensured appropriate learning objectives. Studying and gathering solutions was performed by self study within the group of students using current literature. Afterwards, results were discussed and if necessary corrected by the tutor in accordance with the ERC guidelines.

During the high-fidelity hands on simulator training, students were obliged to solve an advanced CPR-situation by reacting on the condition presented by the high-fidelity manikin. The presented situation was an asystole patient discovered during ward round. According to the above mentioned PBL-tutorial process, analysing of the manikins condition and performing CPR was performed within the group with the possibility to correct each other. A tutor introduced and observed the case but was not allowed to disrupt or guide the participants for correct CPR during the scenario. To account for a realistic environment the simulator was placed in a simulated general hospital ward. A debriefing was done afterwards.

During the practical scenarios, both groups had free access to a standardized set of airway management tools and a manual defibrillator (LIFEPAK 10, Physio-Control, Redmond, USA) without automated feedback on CPR quality.

### CPR skill assessment and data collection

Two hours after the training, participants were randomly divided into pairs. It was ensured that each pair did not complete the initial training together but had been randomized to the same study-group.

For data collection, four identical patient rooms on a simulated general hospital ward were equipped with a CPR-training manikin (Resusci Anne, Laerdal Medical GmbH, Puchheim) in a cardiac arrest scenario. The participants had access to a standardized set of drugs, airway management tools and a defibrillator. The time from entering the room, to first contact, to assessment and initiation of CPR was documented accordingly using video recording. The evaluation of the video documentation concerning specific time points was performed by an external person having not participated in teaching or data analysis using a checklist based evaluation protocol. The manikin data was collected by Laerdal PC SkillReporting System (Fa. Laerdal Medical GmbH, Puchheim). Each scenario and video-documentation lasted for 5 minutes.

### Follow-up

An unannounced follow-up session was conducted six months later during a regularly scheduled course. The same scenario as described above was instituted. The investigators were blinded to participant’s previous group allocation. Students were again divided into groups of two that matched the initial study group assignment, but were different to the first skill assessment.

### Evaluation of guideline conform CPR

The primary endpoint “Guideline conform CPR” was defined as initial evaluation of the manikin’s condition, including breath control and call for help followed by sufficient chest compression. Sufficient chest compression was defined as a sequence of at least ten compressions in a row with a minimum compression depth of 45 mm and a frequency between 90 and 120 compressions per minute.

Besides the primary endpoint, a number of secondary endpoints were evaluated including hands-off time, and time from start of the scenario to the first sufficient chest compression. Other secondary endpoints were time to first ten sufficient compressions in a row as well as quality and quantity of ventilation.

### Statistical analysis

All data are presented as mean ± standard deviation (SD) or Median and 25%/75% quartiles for non-normally distributed data. Statistical analysis was performed with SPSS statistical package (version 20.0; IBM, Armonk, USA). Normal distribution of data was assessed using a Kolmogorov-Smirnov-test. Wilcoxon signed-rank test or Mann-Whitney-U test were used for non-normally distributed data as appropriate for comparisons between or within groups. Student’s t test were used after testing for normal distribution. Categorical data was analysed by Chi square test. Effect sizes were calculated as Hedges’ g (bias-corrected standardized mean difference) with values of 0.2 indicating small and values of 0.8 indicating large effects. [19] A *p* value < 0.05 was considered statistically significant with Holm-Bonferroni correction for multiple testing if applicable.

## Results

One hundred twelve of 127 fourth year students participated in the module of the anaesthesiology course and were enrolled in the study. One hundred eleven complete sets of questionnaires were returned. Fifty four students were assigned to the control group and 58 to the intervention group. Due to a lack in data recording, 3 manikin data sets in the control group and 2 in the intervention group could only partially be evaluated.

Six month later 50 of the previously enrolled students participated in the follow-up scenario, of which 22 were included in the control group follow-up (CFU) and 28 in the intervention group follow-up (IFU).

Due to the group approach in our study, manikin data were analysed for each group of two participants. Therefore, the number of analysed manikin data sets was 27 respectively 29 for the initial data collection and 11 respectively 14 for the follow up.

Participants’ demographical data, previous medical education, period of time since the last CPR-training and experience in real-life CPR scenarios were similar amongst both groups (Table 1).

**Table 1:** Demographic data and previous experience in CPR

	Control	Intervention
N (Students)	54	58
Age (in years)	24.9 ± 3.9	24.0 ± 2,6 (n.s.)
Gender	37,1% male 62,9% female	32.8% male 67.2% female
last CPR training (in Months)	15.2 ± 8.7	13.6 ± 8.2 (n.s.)
Experience in real CPR	27.8%	22.4% (n.s.)
Previous medical education	20.4%	20.7% (n.s.)

Values are presented as mean +/− SD, percentage of participants or numbers of participants

### Compression quality

**Fig. 1:**
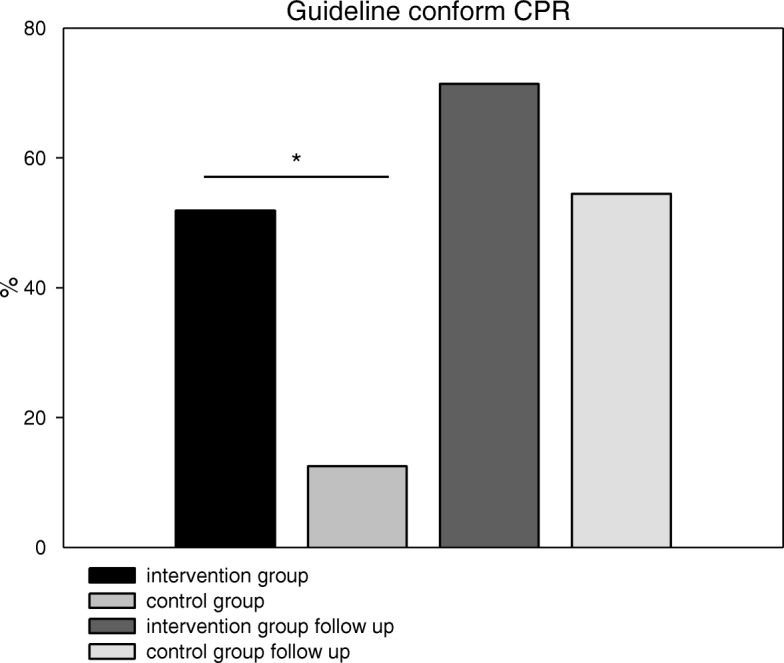
Percentage of ERC-Guideline conform CPR. Intervention vs. control group (Intervention group 28 out of 54, 51.9%; control group 6 out of 48, 12.5%), Follow-up after six months (Intervention group 20 out of 28, 71.4%; control 12 out of 22, 54.5%); (* = *p* < 0.05)

“Guideline conform CPR” was performed significantly more in the intervention group as compared to the control group (51.9% vs. 12.5%, *p* = 0.007; Fig. 1). During the follow-up session, no differences in guideline conform CPR between groups (71.4% vs. 54.5%, *p* = 0.55; Fig. 1) were found.

The percentage of sufficient compressions meeting the criteria of at least 45 mm depth and 90–120 bpm showed no significant difference between the groups in both the 2 h and the follow-up skills assessment (intervention: 42,9 ± 34% vs. control: 41,2 ± 33% *p* = 0.98; IFU: 41,5 ± 31% vs. CFU: 45,1 ± 37% *p* = 0.93). The number of consecutive sufficient compressions was also not different between groups (intervention: 13.5(1.9/27.7) vs. control: 8.0(1.3/14.8), *p* = 0.37; IFU: 9.7(3.6/21.1) vs. CFU: 8.6(3.6/25.1), *p* = 0.99; Table 2).

**Table 2:** Compression times and compression quality

	intervention group	control group	intervention follow-up	control follow-up
Compression frequency (bpm)	112(105/125)	125(104.5/133) (p = 0,17)	118(97/120)	114(102/128) (*p* = 0.525)
Compression depth (mm)	43.9 ± 1.8	42.8 ± 1.9 (*p* = 0.558)	44.3 ± 8	43.9 ± 11 (*p* = 0.880)
Hands-off time (%)	24.0(23.0/26.7)	28.3(24.3/31.7) (*p* = 0.007)	23.7(19.3/26.6)	31.0(25.0/33.7) (*p* = 0.006)
Sufficient compressions (%)	42.9 ± 34	41.2 ± 33 (*p* = 0.984)	41.5 ± 31	45.1 ± 37 (*p* = 0.928)
Sufficient compressions in a row	13.5(1.9/27.7)	8.0(1.3/14.8) (p = 0.37)	9.7(3.6/21.1)	8.6(3.6/25.1) (p = 0.99)
Time to first sufficient compression (sec.)	41.2(33.0/53.5)	32.6(21.4/77.7) (p = 0.12)	41.2(33.8/57.0)	38.4(31.6/120.7) (p = 0.85)
Time to first 10 sufficient compressions (sec.)	52.3(39.3/72.8)	109.6(42.6/158.2) (p = 0.03)	46.9(40.6/103.5)	43.0(34.5/59.8) (p = 0.52)

Values are presented as mean +/− SD or median and (25/75) quartiles

**Fig. 2:**
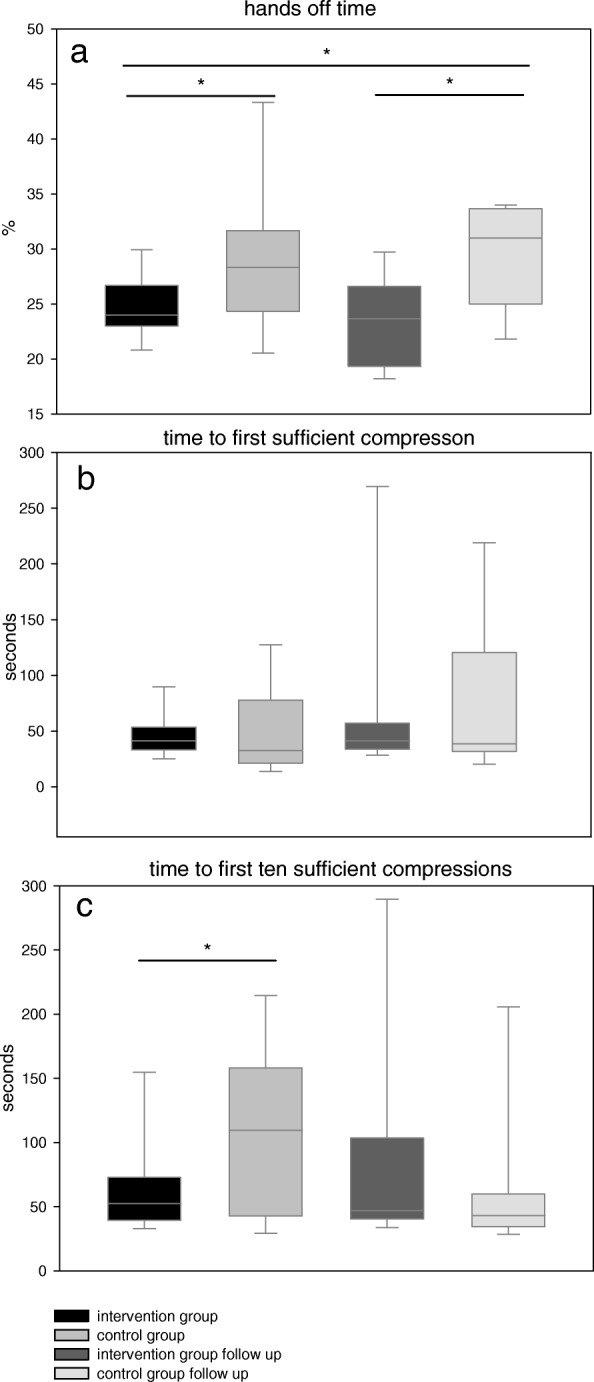
Compression quality. **a** Percentage of hands off time during treatment (intervention group 24.0(23.0/26.7)%, control group 28.3(24.3/31.7)%;Follow-up: intervention group 23.7(19.3/26.6)%, control group 31.0(25.0/33.7)%) **b** Time from beginning to first sufficient compression (intervention group 41.2(33.0/53.5)sec., control group 32.6(21.4/77.7)sec.; Follow-up: intervention group 41.2(33.8/57.0)sec., control group 38.4(31.6/120.7)sec.) **c** Time from beginning to first ten sufficient compressions in a row (intervention group 52.3(39.3/72.8)sec., control group 109.6(42.6/158.2)sec.; Follow-up: intervention group 46.9(40.6/103.5)sec., control group 43.0(34.5/59.8)sec.); (* = p < 0.05)

The intervention group had a significantly lower percentage of interruptions during CPR (hands off time 24.0(23.0/26.7)% vs. 28.3(24.3/31.7)% *p* = 0.007, *g* = 1.55). This was reproduced during the follow-up (IFU: 23.7(19.3/26.6)% vs. CFU: 31.0(25.0/33.7)% *p* = 0.00*6*, *g* = 1.88; Fig. 2a). There was no difference between the groups regarding the time to first sufficient compression in the skills assessment (41.2(33.0/53.5)sec. vs. 32.6(21.4/77.7)sec., *p* = 0.12) and follow-up (41.2(33.8/57.0)sec. vs. 38.4(31.6/120.7)sec., *p* = 0.85; Fig. 2b).

The intervention group reached the target of first ten consecutive sufficient compressions significantly faster than the control group (52.3(39.3/72.8)sec. vs. 109.6(42.6/158.2)sec., *p* = 0.03; Fig. 2c). This difference was not replicated during the follow-up session (IFU: 46.9(40.6/103.5)sec.; CFU: 43.0(34.5/59.8)sec., *p* = 0.52).

The mean compression frequency tended to be non-significantly higher in the control group (intervention: 112(105/125)bpm vs. control: 125(104.5/133)bpm, *p* = 0.17), with the intervention group being closer to the recommended frequency of 100/min. During the follow-up, no difference between groups was revealed (IFU: 118(97/120)bpm vs. CFU: 114(102/128)bpm, *p* = 0.52). Compression depth at skill-assessment and follow-up were similar (intervention: 44 ± 8 mm vs. control 43 ± 9 mm, *p* = 0.56; IFU: 44 ± 8 mm vs. CFU: 44 ± 11 mm, *p* = 0.88; Table 2).

### Ventilation quality

**Fig. 3:**
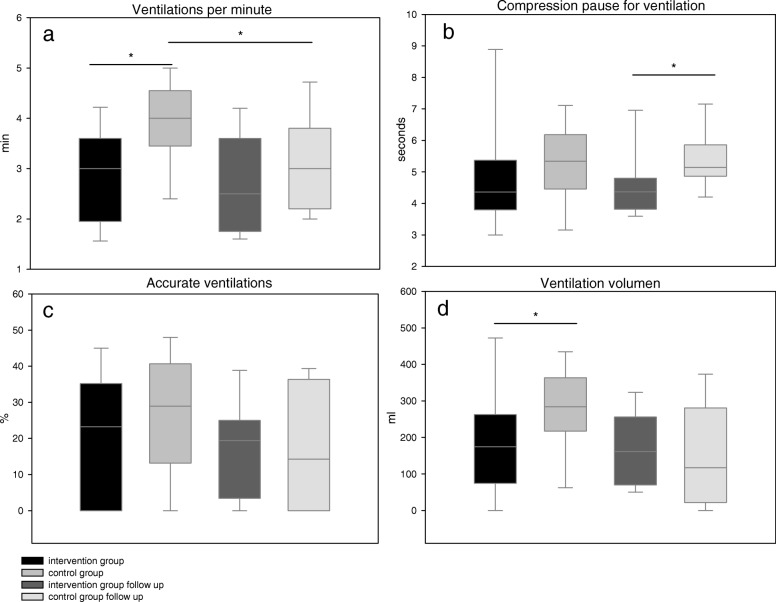
Ventilation quality. **a** Mean ventilations per minute (intervention group 2.8 ± 0.8 min^− 1^,control group 3.9 ± 0.9 min^− 1^; Follow-up: intervention 2.7 ± 1.0 min^− 1^, control group 3.2 ± 1.0 min^− 1^) **b** Mean compression pause for ventilation (intervention group 4.4(3.8/5.4)sec., control group 5.3(4.5/6.1)sec.; Follow-up: intervention 4.4(3.8/4.8)sec., control group 5.1(4.9/5.9)sec.) **c** Percentage of accurate ventilations (intervention group 23.2(0/35.2%)%,control group 29.0(13.2/40.7)%; Follow-up: intervention 19.4(3.4/25.0)%, control group 15.9(0/36.4)%) **d** Mean ventilation tidal volume (intervention group 185 ± 155 ml, control group 275 ± 115 ml; Follow-up: intervention 169 ± 97 ml, control group 150 ± 141 ml); (* = p < 0.05)

Both groups tended to administer a lower ventilation rate than recommended by the ERC guidelines. The highest rate of ventilations per minute (VPM) was measured in the control group (control: 3.9 ± 0.9 VPM, intervention: 2.8 ± 0.8 VPM, *p* = 0.001; IFU: 2.7 ± 1.0 VPM, CFU: 3.2 ± 1.0 VPM, *p* = 0.7) with ongoing significant difference compared to CFU (*p* = 0.03; Fig. 3a). In the intervention group, the compression breaks for ventilation tended to be shorter (4.4(3.8/5.4)sec. vs. 5.3(4.5/6.1)sec., *p* = 0.14) and were significantly shorter during the follow-up (IFU: 4.4(3.8/4.8) sec. vs. CFU: 5.1(4.9/5.9) sec., *p* = 0.05; Fig. 3b). The percentage of ventilations with a sufficient air-flow showed no difference between groups (intervention: 23.2(0/35.2)% vs. control: 29.0(13.2/40.7)%, *p* = 0.37; IFU: 19.4(3.4/25.0)% vs. CFU: 15.9(0/36.4)%, *p* = 0.73; Fig. 3c). The mean tidal volume was significantly lower in the intervention group (186 ± 155 ml vs. 275 ± 115 ml, *p* = 0.03). The follow-up showed no difference between intervention and control group (169 ± 97 ml vs. 150 ± 141 ml, *p* = 0.539 Fig. 3d).

### Self-assessment by questionnaire

All students sensed the CPR-algorithm as taught completely and memorably after the training, irrespectively of the teaching technique they participated in (intervention: 5.3 ± 0.8 vs. control: 5.4 ± 0.8, *p* = 0.36).

**Fig. 4:**
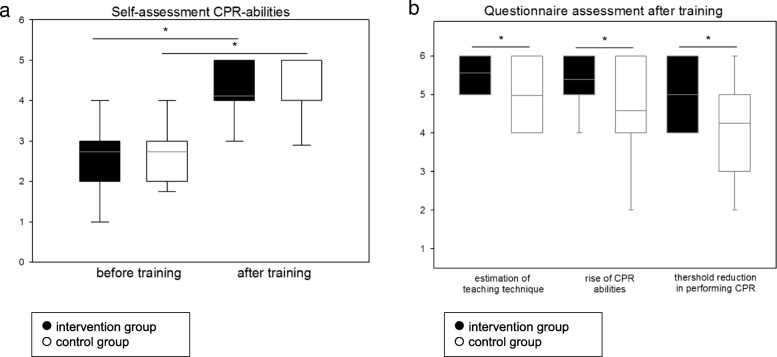
Questionnaires. **a** Students change in self-assessment in CPR skills during the training (intervention 2.7 ± 1.0 vs. 4.1 ± 0.7; control 2.7 ± 1.0 vs. 4.0 ± 0.9) **b** Questionnaire assessment after training in adequacy of the teaching technique (intervention: 5.0 ± 1.0 vs. control: 5.6 ± 0.8), self-estimation of increase in CPR abilities (intervention: 4.6 ± 1.4 vs. control: 5.4 ± 0.9) and threshold reduction in performing CPR (intervention: 4.3 ± 1.5 vs. control: 5.1 ± 1.1) between both groups; (* = p < 0.05)

After the training, self-assessment of CPR-competence increased (intervention: 2.7 ± 1.0 vs. 4.1 ± 0.7, *p* < 0.001; control: 2.7 ± 1.0 vs. 4.0 ± 0.9, *p* < 0.001; Fig. 4a), with no difference between groups. Students in the control group estimated their threshold reduction to perform CPR significantly higher (intervention: 4.3 ± 1.5 vs. control: 5.1 ± 1.1, *p* < 0.001; Fig. 4b). Also the self-estimated improvement in CPR abilities due to the training (intervention: 4.6 ± 1.4 vs. control: 5.4 ± 0.9, *p* < 0.001; Fig. 4b) and the estimated effectiveness of the teaching technique (intervention: 5.0 ± 1.0 vs. control: 5.6 ± 0.8, *p* < 0.001; Fig. 4b) were higher in the control group.

## Discussion

The aim of the study was to investigate, whether PBL combined with high-fidelity full-scale simulator training improve short- and long-term CPR skills, according to the ERC/ILCOR guidelines, compared to a classical, hands on tutor-guided CPR training. Our data demonstrate that students in the high-fidelity simulated intervention group were more often able to perform sufficient guideline conform CPR compared to the control students. After 6 months, there were still favorable effects of the interventional teaching approach but without significant differences in the primary endpoint.

The primary purpose of performing CPR is the maintenance of cerebral blood flow, as emphasized by the ERC guidelines, which changed the chest compression/ventilation ratio from 15/2 to 30/2 in adults in 2005. [20, 21] The 2010 and 2015 guideline-revisions underline the importance of minimal interruptions, sufficient depth and frequency of compressions. [22] Former investigations studying CPR education and subsequent quality used single endpoints such as compression depth or frequency. [23, 24] In contrast, we used “Guideline conforming CPR” as a more comprehensive definition of successful CPR as the study’s primary endpoint.

To represent this, a holistic approach of evaluating the CPR guidelines’ requirements of compression quality is reasonable to determine between sufficient and insufficient CPR.

Kern et al. showed, that every interruption of chest compressions leads to an immediate loss of coronary perfusion pressure and that approximately five to ten successfully performed compressions in a row are necessary to re-establish the pressure to previous levels. [25] Hence, only high quality compressions without interruptions can be rated as a potential benefit. Furthermore, to ensure that the participant was aware of the patient’s condition and to make our results more applicable to real CPR situations, we hypothesized, that without performing a prior diagnostic bloc, CPR in an artificial scenario cannot be considered successful.

In meeting the requirement of patient evaluation followed by correctly indicated sufficient chest compressions as primary endpoint, the intervention group outperformed the control group fourfold (51.9% vs. 12.5%). This difference was not carried forward to the folow up measurement, where both groups reached similar improved results.

The short term superiority of the intervention group may be explained by the PBL-tutorial process, which was shown in a retrospective study on CPR-education of nurses. [26] The combination of theoretical and practical PBL-process, with peer guidance and self-developed problem solution may lead to better results in a situation, where theoretically and practically skills are crucial. Evaluating the improved performances in both groups in the follow up scenario, we cannot conclude, that the short term superiority of the PBL-process can be responsible for long lasting effects. Due to the also improved results in the control group, we cannot exclude, that a highly motivation in medical students for reaching a good CPR-performance after the first training may lead to the good overall results in both groups.

Another important factor of patient’s survival and neurological outcome [1], is the establishment of sufficient perfusion pressure and blood flow during CPR as early as possible. This was reached 57 s faster in the intervention group from the beginning of the scenario until the first ten sufficient cardio compressions performed in a row.

It is well known, that a delay of the initiation of CPR treatment in a cardiac arrest situation reduces the chance of survival and the outcome of the patient. [27–29] Higher self-confidence of physicians regarding their abilities and optimal training-methods may lead to a more self-confident behaviour and thus to a faster evaluation of cardiac arrest, followed by a faster initiation of CPR. This was supported by research performed by Verplancke et al., who found improved compression and ventilation skills in more self-confident trainees. [10]

In self assessment, both groups in our study estimated their CPR-abilities after the training to be significantly higher compared to the initial evaluation, with no differences between the groups. Despite this, the control group reported a significantly higher self-confidence by estimating their inhibition threshold reduction in performing CPR, as well as the increase of their own CPR-abilities and the competence of the teaching technique they fulfilled. Comparing the higher self-confidence with the less effective performed CPR in the control group contrast to the findings of Verplancke et al.. This may be explained by a phenomenon termed illusory superiority, a well-known effect where a lack of knowledge leads to overestimation of competence. [30] It is assumable that a more realistic environment and a teaching technique closer to the needs of real performed CPR leads to a more realistic self-assessment in the intervention group, whereas the control group overestimated their abilities due to a lack of knowledge.

Beside initiating CPR, limiting interruptions during CPR is crucial for maintaining blood flow. As emphasized by the ERC-guidelines [22] and Kern et al. [25], hands-off time is an important component for maintaining blood flow. Compared to an evaluation of professional first responders [31], both groups in our study maintained a more continuously chest compression shown by lower percentage of hands off time, where the intervention group with 24% outperformed the control group significantly.

Some other particular target values of sufficient CPR showed relevant differences between both groups.

The guidelines recommend a rate of approximately 100 compressions per minute without exceeding 120 bpm. [22] A recently published meta-analysis reported beneficial compression rates of 85-120 bpm but with a reduced survival in exceeding 120 bpm. [32] Based on this, all compressions between 90 and 120 bpm were defined in our study as successful. The intervention group reached this goal while students in the control group performed chest compressions at a frequency of approximately 121 bpm (median 125 bpm).

The compression depth reached an average of 43.7 mm over all groups and therewith was lower than the guideline’s requirements [22] but within the range of 40.3 to 55.3 mm, which showed the maximum survival in a recently published study. [33]

In both groups, we found a serious lack of ventilation frequency and ventilation volume compared to the recommendations. [22] Other works evaluating medical students and health care professionals performance on CPR-manikins showed similar results of low ventilation frequency and volume. [24, 34] Several theories might explain these results: Sufficient ventilation could be a difficult goal to achieve for the untrained and airway management skills are obviously more advanced than the skills needed for delivering adequate chest compressions. These findings might also be a result of observed little efforts by the trainees to optimize ventilation, possibly caused by the guideline related strong focus placed on performing adequate chest compression and minimizing hands-off time. This theory is supported by results of Jones et al., who showed an increase in compression frequency in 2010 CPR guideline educated students as compared to 2005 guideline educated students. [35]

Therefore, despite the undoubted necessity of adequate chest compression, the teaching of airway management in CPR-situations for health care professionals should not be neglected.

In contrast to previously published results showing no difference in long-term retention of theoretical CPR knowledge between traditional and high-fidelity educated students [13], our work demonstrated after 6 month some relevant differences in solitary measurements. The follow up still reveals a significantly lower hands-off time and a reduced compression pause for ventilation in the interventional group. However, the initial significant difference between groups in time to achieve the first ten sufficient compressions was not carried forward to the follow up, due to an improvement in the control group. The improvement of particular endpoints after 6 month in the control group may lead to a loss of measurable differences between the two analysed teaching techniques which is maybe explained by a over representation of highly motivated and skilled students during the follow up.

### Limitations

Different learning experiences among the participants during the 6 months before the unannounced follow-up evaluation were not measured. Further, according to the university regulations, the follow-up took place during a voluntary anaesthesiology course, where only 45% of the initial participants took part. This may have led to a selection bias with an over representation of highly motivated and above average skilled students in both groups.

Further, to maintain a maximum of blinding, we tested multiple students at the same time with the same CPR-scenario in four rooms, while others were still in course core lectures. We cannot exclude communication between already tested and still to be tested students during data collection.

## Conclusion

Our study shows that even directly after an intensive CPR training, the guideline requirements on CPR are hard to achieve.

Hence, the best possible learning strategy should include a broad expertise with a significant emphasis on long-term skill retention. PBL combined with a high-fidelity CPR-training leads to a measurable short-term increase in initiating sufficient CPR, with some long-lasting effects. In contrast, the classical education leads to an overestimation in self-confidence most likely owing to a lack of knowledge, which seems to be crucial to avoid.

Therefore, a periodic self-guided training, which can be achieved using high-fidelity CPR-manikins appears to be a promising way to reach the aim of maintaining persistent sufficient CPR skills and should be implemented in every medical student’s training.

## Additional file

Additional file 1: Self-assessment by questionnaire. Initial self-assessment by questionnaire for empirical data collection and self-perception of personal CPR-skills followed by a second set of questionnaires covering the training and time-dependent self-perception of the students’ CPR-skills. (DOC 28 kb)

## Data Availability

The datasets supporting the conclusions of this article are included within the article and its additional files. Files containing all raw data are available from the corresponding author on request. The questionnaires used are attached as a supplementary file.

## Abbreviations

ALS: Advanced live support
BLS: Basic live support
CFU: Control group follow-up
CPR: Cardiopulmonary resuscitation
IFU: Intervention group follow-up
PBL: Problem-based learning
SEM: Standard error of the mean
VPM: Ventilations per minute
