# Supplementary material for: Combination of problem-based learning with high-fidelity simulation in CPR training improves short and long-term CPR skills: a randomised single blinded trial
Source: BMC Med Educ. 2019 May 31;19:180. doi: 10.1186/s12909-019-1626-7 (PMC6544917; doi:10.1186/s12909-019-1626-7)
Supplement: Supplementary file 1 — Self-assessment by questionnaire. Initial self-assessment by questionnaire for empirical data collection and self-perception of personal CPR-skills followed by a second set of questionnaires covering the training and time-dependent self-perception of the students’ CPR-skills. (DOC 28 kb) [file 12909_2019_1626_MOESM1_ESM.doc]

**Evaluation of the cardiopulmonal-resuscitation (CPR)-training**

matriculation number ____________

Please rate the questions below between 1 (not at all appropriate) and 6 (absolutely appropriate)

1. The CPR-algorithm was taught complete and memorable ___

2. The studying technique is suitable for CPR-education ___

3. My practical skills in performing CPR have risen due to the course ___

4. My inhibition threshold in performing real CPR is reduced due ___
 to the course

5. Estimate your actual abilities in performing CPR ___
 1 (no abilities) – 6 (perfect abilities)

**Self-assessment cardiopulmonal-resuscitation (CPR)**

matriculation number _____________

How old are you? ___ Years

What is your gender? 🞏 M 🞏 F

When was your last CPR-training ___Month 🞏never

Have you performed CPR in a real situation before 🞏 yes 🞏 no

Do you have a medical education 🞏 yes 🞏 no
(e.g. paramedic, nurse)

Estimate your actual abilities in performing CPR ___
 1 (no abilities) – 6 (perfect abilities)
